# Single-Plex Quantitative Assays for the Detection and Quantification of Most Pneumococcal Serotypes

**DOI:** 10.1371/journal.pone.0121064

**Published:** 2015-03-23

**Authors:** Fuminori Sakai, Sopio Chochua, Catherine Satzke, Eileen M. Dunne, Kim Mulholland, Keith P. Klugman, Jorge E. Vidal

**Affiliations:** 1 Hubert Department of Global Health, Rollins School of Public Health, Emory University, Atlanta, Georgia, United States of America; 2 Pneumococcal Research, Murdoch Childrens Research Institute, The University of Melbourne Department of Paediatrics at the Royal Children’s Hospital, Parkville, Victoria, Australia; 3 Department of Microbiology and Immunology at the Peter Doherty Institute for Infection and Immunity, The University of Melbourne, Victoria, Australia; 4 London School of Hygiene & Tropical Medicine, London, United Kingdom; USDA-ARS-ERRC, UNITED STATES

## Abstract

*Streptococcus pneumoniae* globally kills more children than any other infectious disease every year. A prerequisite for pneumococcal disease and transmission is colonization of the nasopharynx. While the introduction of pneumococcal conjugate vaccines has reduced the burden of pneumococcal disease, understanding the impact of vaccination on nasopharyngeal colonization has been hampered by the lack of sensitive quantitative methods for the detection of >90 known *S*. *pneumoniae* serotypes. In this work, we developed 27 new quantitative (q)PCR reactions and optimized 26 for a total of 53 qPCR reactions targeting pneumococcal serotypes or serogroups, including all vaccine types. Reactions proved to be target-specific with a limit of detection of 2 genome equivalents per reaction. Given the number of probes required for these assays and their unknown shelf-life, the stability of cryopreserved reagents was evaluated. Our studies demonstrate that two-year cryopreserved probes had similar limit of detection as freshly-diluted probes. Moreover, efficiency and limit of detection of 1-month cryopreserved, ready-to-use, qPCR reaction mixtures were similar to those of freshly prepared mixtures. Using these reactions, our proof-of-concept studies utilizing nasopharyngeal samples (N=30) collected from young children detected samples containing ≥2 serotypes/serogroups. Samples colonized by multiple serotypes/serogroups always had a serotype that contributes at least 50% of the pneumococcal load. In addition, a molecular approach called S6-q(PCR)^2^ was developed and proven to individually detect and quantify epidemiologically-important serogroup 6 strains including 6A, 6B, 6C and 6D. This technology will be useful for epidemiological studies, diagnostic platforms and to study the pneumobiome.

## Introduction

*Streptococcus pneumoniae* (the pneumococcus) is a major human pathogen that causes diseases, such as otitis media, pneumonia, septicemia and meningitis in children, the elderly and immune compromised patients [1–3]. There are more than 90 pneumococcal capsular types (serotypes) although a subset of serotypes are responsible for most cases of pneumococcal disease (PD) [4, 5]. Globally each year, the pneumococcus causes 15 million cases of serious disease, which lead to approximately 1 million deaths in children [5–7]. Vaccination with pneumococcal conjugate vaccines (PCVs), the basis for which is induction of a protective antibody response against the bacterial polysaccharide capsule, has dramatically reduced the global burden of PD [4, 8]. Licensed conjugated vaccines in the USA previously contained seven serotypes (4, 6B, 9V, 14, 18C, 19F, 23F), but since 2010 have contained 13 (PCV7 plus 1, 3, 5, 6A, 7F and 19A) of the ∼94 known pneumococcal serotypes.

A prerequisite for PD is colonization of the human nasopharynx and persistence in this niche, known as carriage. Pneumococcal carriage commonly occurs in early childhood [9, 10]. Before PCVs were introduced, the most prevalent serotypes implicated in PD and carried in the nasopharynx belonged to vaccine types [4, 8]. In the post-PCV era, studies have consistently reported that carriage of vaccine types is decreasing whereas non-vaccine types are now increasingly detected in nasopharyngeal (NP) samples, a phenomenon called serotype replacement [4, 9, 11].

For nearly 50 years, studies of pneumococcal carriage have been based on bacterial culture [12, 13]. Culture methods isolate one or more colonies from NP samples, for which the serotype is identified using serotype-specific antibodies, known as Quellung reaction or capsular swelling reaction [14, 15]. The serotype can be alternatively investigated by a multiplex PCR approach [16, 17].

Multiple carriage of pneumococcal strains has been investigated with modified culture methods including conventional Quellung typing of at least five colonies [18], or latex agglutination of a sweep of colonies from a plate culture [19, 20]. Other methods isolate DNA directly from the NP sample, or after an additional enrichment step, and utilize different platforms such as multiplex PCR approaches [21–23], a microarray platform [19] or a MassTag PCR assay [24].

Recently, a very high prevalence of multiple pneumococcal carriage was published by Turner et al., by using both a sweep-latex agglutination method and microarray studies to demonstrate that 43% or 48.8%, respectively, of NP swabs from Thai children carried more than one pneumococcal serotype [19]. A similar prevalence of multiple serotype carriage (∼40%) was observed recently in Spain utilizing a combination of Quellung reactions and latex serotyping along with multiplex PCR reactions [23]. Limitations of these methods is that they are not quantitative although the microarray provides relative proportions of each serotype present in a sample and may be quantitative when used together with a *lyt*A quantitiatve PCR (qPCR) [19, 25].

Two different groups have published a series of real-time PCR reactions for the detection of more than 20 pneumococcal serotypes in single-plex reactions [22, 26] or a multiplex reactions format [27]. Using real-time PCR, Azzari et al. detected multiple carriage in ∼60% of NP samples collected from children in Italy [22]. A multiplex real-time PCR approach developed by Pimenta et al. (2013) was shown to be specific for the target pneumococcal serotypes or serogroups [27].

We have developed in this work protocols to optimize and validate quantitative real-time PCR assays for pneumococcal serotyping and have developed new qPCR assays that, together with published sequences, allow for the detection and for the determination of the bacterial load of 72 pneumococcal serotypes/serogroups. Given the quantitative nature of these single-plex assays, we hereafter referred to these as qPCR to differentiate quantitative assays from highly-sensitive, qualitative, real time reactions [22, 27]. As a proof-of-concept, we tested 30 pneumococcal-positive NP samples and identified at least one serotype in all tested NP samples. Half of those samples contained more than one serotype, and we identified up to 5 different serotypes in a single sample. These quantitative assays identified serotypes for whom the serotype-specific proportion was as little as 0.01% of the total pneumococcal load, i.e., as a proportion of all combined serotype loads.

## Material and Methods

### Nasopharyngeal samples

NP samples (n = 30) were previously collected from children aged ≤ 24 months in Papua New Guinea (n = 14), South Africa (n = 6), Bangladesh (n = 4), Fiji (n = 2), Kenya (n = 2) and The Gambia (n = 2) as part of the PneuCarriage project [28]. Samples were collected and stored at -80°C in skim-milk-tryptone-glucose-glycerin (STGG) transport medium [29] according to WHO guidelines [14, 30]. Samples underwent one freeze-thaw for aliquoting purposes, and 60 μl aliquots were shipped on dry ice and stored at -80°C prior to testing by qPCR.

### Bacterial strains utilized in this study

*S*. *pneumoniae* serotypes utilized in this study were obtained either from the Statens Serum Institute (SSI) or had been previously typed by the Quellung reaction at the Centers for Disease Control and Prevention (CDC) and kindly provided by Dr. Lesley McGee and Dr. Bernard Beall, or isolated in our laboratories and Quellung typed. Pneumococcal strains include the following serotypes: 1, 3, 4, 5, 6A, 6B, 6C, 6D, 7A, 7B, 7C, 7F, 8, 9A, 9L, 9N, 9V, 10A, 10B, 11A, 11D, 11F, 12A, 12B, 12F, 13, 14, 15A, 15B, 15C, 15F, 16F, 18B, 18C, 19A, 19B, 19F, 19“F” (atypical), 20, 21, 22A, 22F, 23A, 23B, 23F, 24A, 25A, 25F, 27, 29, 31, 33A, 33B, 33D, 33F, 34, 35A, 35B, 35C, 35F, 36, 38, 39, 40, 41A, 41F, 42, 43, 45, 46, 47A, 47F.

Streptococci naturally found in the nasopharynx and other anatomic sites were also utilized to validate *S*. *pneumoniae* serotype-specific qPCR assays and included: *S*. *infantis*, *S*. *oralis*, *S*. *anguinosus*, *S*. *intermedius*, *S*. *sobrinus*, *S*. *pseudopneumoniae*, *S*. *mitis*, *S*. *parasanguinis*, *S*. *australis*, *S*. *mutans*, *S*. *peroris*, *S*. *oligofermentans*, *S*. *intestinalis*, *S*. *vestibularis*, *S*. *cristatus*, *S*. *salivarius*, *S*. *gordonii*, *S*. *sanguinis*, *S*. *sinensis* and *Dolosigranulum pigrum* [31, 32]. Reference, genome-sequenced, TIGR4 (GenBank accession # Z_AAGY00000000) [33] was utilized to prepare DNA standards for the *lyt*A qPCR assay [25].

### DNA extraction from reference strains and nasopharyngeal samples

DNA from bacterial cultures and nasopharyngeal samples was purified using the QIAamp DNA mini kit (Qiagen) as detailed elsewhere [21, 31, 34]. DNA from bacterial cultures, or from NP samples, was eluted in a final volume of 100 μl. Quality and quantification of DNA preps obtained from bacterial cultures was further evaluated using the Nanodrop system (Nanodrop Technologies, Wilmington, DE).

### Preparations of DNA standards for quantitative (qPCR) assays

Purified DNA from *S*. *pneumoniae* serotype control strains was adjusted to a concentration of 1 ng/μl in TE buffer (10mM Tris-HCl, 1mM EDTA, pH8.0) and immediately stored at -80°C until use. Standards for quantification purposes were prepared within an hour before reactions were performed by serial dilution of a 1 ng/μl aliquot in TE buffer to a final concentration of 100 pg/μl, 10 pg/μl, 1 pg/μl, 100 fg/μl, 50 fg/μl, or 5 fg/μl of pneumococcal DNA. Given the 2.1608 Mb genome size of TIGR4 [33], these standards corresponded to 4.29x10^5^, 4.29x10^4^, 4.29x10^3^, 4.29x10^2^, 4.29x10^1^, 2.14x10^1^, or 2.14 genome equivalents, respectively. Standards prepared using this protocol obtained an efficiency >90% throughout the study (not shown).

### qPCR studies

The total density of *S*. *pneumoniae*, reported as CFU/ml, was determined by a qPCR assay targeting the *lyt*A gene, essentially as described previously [25]. Reactions were performed utilizing Platinum Quantitative PCR Super Mix-UDG (life technology, USA) and 2.5 μl of pure DNA as template. Final concentration and sequences of the following primer and probe were utilized: forward primer (5’-ACGCAATCTAGCAGATGAAGCA-3’; 100 nM), reverse primer (5’-TCGTGCGTTTTAATTCCAGCT-3’; 100 nM), and probe (5’-FAM-TGCCGAAAACGCTTGATACAGGGAG-3-BHQ1; 100 nM). Details of cycling conditions were reported in [25]. Serotype-specific reactions were performed in 10 μl reactions containing 1x SsoAdvanced Universal Probes Supermix (Bio-Rad, Hercules, CA), serotype-specific primers and probe at a concentration listed in Table 1 and S1 Table, and 1 μl of DNA as template. Serotype specific qPCR reactions were conducted using: one cycle of 95°C for 2 min; 40 cycles of 95°C for 15 s and 60°C for 30 s. To quantify the molecular bacterial load (CFU/ml), genomic DNA purified from control stains was serially diluted to prepare standards as described earlier. These standards were run along with DNA from NP samples in a CFX96 real time PCR-detection system (Bio-Rad, Hercules CA) and CFU/ml were calculated using the software Bio-Rad CFX manager.

**Table 1:** Primers and probes designed and validated in this study.

Serotype		Sequence*	Target region	Accession No.	Position	Size (bp)	Limit of detection (fg)	Concentration (nM)
6CD	Forward	CAATCAGGCAGTTCTTTTCTCG	*wciNbeta*	EF538714	7319–7340	133	5	500
	Reverse	ACCTGACTCACCATCAATAACC			7430–7451			500
	Probe	AAATGGGAGGGCTTTGGATTGGC			7341–7363			200
7BC/40	Forward	TCCAGATATAGTCATTCCCAATCAG	*wcxU*	CR931641	10720–10744	147	50	400
	Reverse	AAAGAAGGTAAATCCCATGATGAATT			10841–10866			400
	Probe	TGGTGGGTCAGTAATCGATAATGAGGGA			10798–10825			200
7C	Forward	AGTTTGAGCATAACGGAGCG	*wchF*	CR931642	7010–7029	132	5	200
	Reverse	GAGCTTCATCCTTATTTTCCTTAGC			7117–7141			200
	Probe	CTCGAGCTGGACCAATATTCGGAACAT			7046–7072			200
9LN	Forward	CGTGGAATTTTCTATACTGCAATAGG	*wzx*	CR931646	11762–11787	115	5	500
	Reverse	CTACTGCTACGATACCATATTCTACAG			11850–11876			500
	Probe	CAGCAATTCTTAGCCGGATTCTCTCACC			11823–11850			200
10A	Forward	AGAGGCCCTAAGAAAAGATTCG	*wcrD*	CR931649	10268–10289	137	5	400
	Reverse	CCCAGTCATCCCCATCAATAAC			10383–10404			400
	Probe	TGTTGAGCCATGACCTCCATTTTCCT			10315–10340			200
10B	Forward	AAATATGAGATTGGTAAGGAATATTCTGG	*wcrD*	CR931650	10237–10265	116	5	400
	Reverse	GTCTTTTCACTTAAACGAATTCCATTC			10326–10352			400
	Probe	AACGGATTCCAATGCACTCGGTAACT			10277–10302			200
11F	Forward	TGGTCCAGCTACTTTTATGGC	*wchK*	CR931657	7485–7505	93	5	400
	Reverse	TGATCATTCACATGCTCCCC			7558–7577			400
	Probe	ACTCCAATAGTTGTTCCGAGGCAAAAGA			7525–7552			200
12B	Forward	TTGGTTGCTGATCAAAAGGTC	*wzx*	CR931659	13259–13279	228	5	600
	Reverse	CATTTTTGGAAGTGGAGCTATC			13465–13486			600
	Probe	TCACTTTGATGATTTGGAAAGATTTT			13338–13363			400
13	Forward	AGACTACCATTTTTTGATCAGTTAGATT	*wzy*	CR931661	13163–13190	136	5	500
	Reverse	CAGAAAACATATTTTGTTCATAAATCCATC			13269–13298			500
	Probe	AAGCAGCACTTCCAAGTCGTAATCTACC			13202–13229			300
19“F”	Forward	GTCCTTAGTTCTGGTTATTCGGG	*wzy*	FJ829071	364–386	106	5	400
	Reverse	GGATGAGGAACCGAATCGAAG			449–469			400
	Probe	CCAGTTATGAAGGTGAGCTAACAGTGCG			419–446			300
24A	Forward	CTTGGAGTTGCTAATTATGGGAAG	*wzx*	CR931686	15526–15549	120	5	300
	Reverse	ATCTCTTACACGTGCACACTC			15625–15645			300
	Probe	CTGCGGGTATTTTACGATATGCTGTGCG			15596–15623			200
25AF	Forward	ATACCAACTAGAATCAGCAGGAC	*wcyE*	CR931689	16881–16903	128	5	400
	Reverse	AAATGGAATATCTTTTGATAATTTACTCGC			16979–17008			400
	Probe	CCCGCTGGACTTACTGCAATACTCG			16949–16973			200
27	Forward	AGCGATTTAGCGACTGATATCC	*whaK*	CR931691	7805–7826	79	50	400
	Reverse	TCTCAAAATCGATCTCGCGTG			7863–7883			400
	Probe	TGTGGAAGGCGTTTGAAGGTGACT			7839–7862			200
31	Forward	GCAGAAGTTTTAAGTCACGGAC	*wzy*	CR931695	9303–9324	146	5	500
	Reverse	AGCATTACAGATGTCACTAAGGG			9426–9448			500
	Probe	CCCCCACGTAAAACCGCAAGG			9395–9415			200
33B	Forward	CCTGTTAGTGCACCTGTATTTAAC	*wciN*	CR931699	7069–7092	146	5	500
	Reverse	GCATTCAAAACTCCTTCATCTCC			7192–7214			500
	Probe	TCCTCGATTCGTTGTTCACGCCA			7123–7145			200
33D	Forward	CGTATAGTCTTGCGACATTTCA	*wciN*	CR931701	7325–7346	81	5	400
	Reverse	TTCCACATGCGTTACCTCAC			7386–7405			400
	Probe	TCAAAAAGACCTTGGCAAGAAAGT			7361–7384			200
34	Forward	CGGTGGAGTAGGTCAAGATG	*wzy*	CR931703	8096–8115	144	5	300
	Reverse	GTCTGTTCTCCCCAATATACTGAG			8216–8239			300
	Probe	ACGGAGCGCCAATGTACTTGAATAGTT			8140–8166			200
35AC/42	Forward	GCTTCCCCTTTAGACTATTCGG	*wcrK*	CR931704	11012–11033	108	5	400
	Reverse	AAATGAAATCAAAGTATCACGTATCGA			11093–11119			400
	Probe	TTCAAAATACCCAGGACACCCGTTCA			11062–11087			200
35F/47F	Forward	GTGGTCGTATATACTTGATGAATAAATCG	*wzy*	CR931707	7694–7722	145	5	400
	Reverse	ACATACAAATTATCAACATACAGATAGGTC			7809–7838			400
	Probe	TCCATTCAACTGGTCGTCCGAATAATCC			7741–7768			200
36	Forward	CTTGTCTATTCAGCCCTTCTGG	*wzy*	CR931708	13287–13308	148	5	500
	Reverse	CGCGATTATATTGTAAATTGGGAACT			13409–13434			500
	Probe	TGCCCGCTACAATGAGATACGTTTTCAA			13341–13368			200
39	Forward	CAAAAAAATGAACTAACTCAAATAGTAACG	*wcrG*	CR931711	12614–12643	143	5	300
	Reverse	ATACTGTAATTTTCTTGTTTATTTGCGG			12729–12756			300
	Probe	AAGTCAGGCGTATTCTTCACAAGGGAAA			12644–12671			200
41A	Forward	GCAAATAGATGTATCCCAGTTAACAC	*wciB*	CR931713	7130–7155	114	50	300
	Reverse	GGTAGCTCTTTTGGTTTAATGTCC			7220–7243			300
	Probe	CACGACCGAATAGTCTAGCTTCAAAGGG			7155–7182			200
41F	Forward	TTTTTGGGAGGAAGTGCTTTT	*wzx*	CR931714	14008–14028	134	5	500
	Reverse	AAAACCGCTTTCTCATGATTC			14121–14141			500
	Probe	TTTCTTCTGTGCTAACAGTGGAGA			14030–14053			200
43	Forward	AGAGGCTACATCAAATAGTTGGC	*wzx*	CR931716	16435–16457	150	5	400
	Reverse	GAATCACACCGTAACTTCCAAAG			16562–16584			400
	Probe	TCCAATAGTACTCACCCCTACCGAGC			16471–16496			200
45	Forward	TCTAGCTACTTGACTAAAATATTTGAACTG	*wzy*	CR931718	13743–13772	88	50	400
	Reverse	GACGAGTCGATTTCGCTGTAT			13810–13830			400
	Probe	TAGGGAGCGAGGTCACTAAAAGTCGT			13774–13799			200
46	Forward	CGAAGTTTTTATATCTCTATTGGTTTG	*wzy*	CR931719	9232–9258	133	50	1000
	Reverse	TATCCCAGGAACTGGACGAA			9345–9364			1000
	Probe	TCATTCTTTCTTCAATTCCTTTCTGA			9319–9344			500
47AF	Forward	AGGAATTGGTAGAGAGTTTGTGG	*whaI*	CR931721	8235–8257	121	50	400
	Reverse	GAAAGTTGAACCATCATCCGTC			8334–8355			400
	Probe	CACTTGATGGAATGCCTGCTGCC			8271–8293			200

*Probes were labeled at 5’ with FAM (6-carboxyfluorescein) and at 3’ with BHQ1 (Black Hole Quencher-1).

### Development of qPCR assays to detect and quantify *S*. *pneumoniae* serotypes

A new set of qPCR reactions (primer and probe sequences) was designed *in silico* utilizing sequences of the *cps* locus available in the GenBank (Table 1). We also included sequences of atypical 19F strains, denoted as 19“F”. The cps loci of this variant was recently described having 88% homology with 19A *cps* sequences [35, 36]. To identify serotype-specific target sequences for our qPCR assays, we aligned the *cps* locus of all available *S*. *pneumoniae* serotypes utilizing NCBI-Basic Local Alignment Search Tool (NCBI-BLAST) and the DNASTAR Lasergene software version 11.2.1 (DNASTAR Inc., Madison WI). Identified serotype-specific sequences were further blasted against all organisms in the NCBI database to verify that there was not significant *in silico* homology with other species. Primer and probe were designed utilizing the software from Integrated DNA technologies (http://www.idtdna.com/site), or Primer3plus (http://www.bioinformatics.nl/cgi-bin/primer3plus/primer3plus). Primer and probe sequences were additionally blasted as above mentioned to further verify specificity for the target pneumococcal serotype(s). Primers and probes were synthesized by Sigma-Aldrich Co.

### High-throughput evaluation of specificity of qPCR assays

The specificity of our newly designed assays, and some previously published reactions [22, 26], was evaluated against DNA (100 pg) purified from all *S*. *pneumoniae* serotypes and several nasopharyngeal streptococci. To set up a rapid and high-throughput screening we prepared two DNA reference libraries, in a 96-well microtiter plate platform, containing either DNA from *S*. *pneumoniae* serotypes or DNA from streptococci species. DNA concentrations were adjusted to 40 pg/μl in 150 μl of TE buffer and then added to each well of a 96-well microtiter plate, and which were cryopreserved at -80°C. We first tested specificity for *S*. *pneumoniae* serotypes as follows: the library of pneumococcal DNA was thawed on ice and 2.5 μl (100 pg/reaction) were transferred with a multi-channel pipette to a qPCR plate containing the reaction cocktail of the serotype-specific qPCR assay to be tested. These real-time reactions were performed in a CFX96 real time PCR-detection system (Bio-Rad, Hercules CA). A typical positive/specific reaction showed a Ct value between 20–30. Once specificity for the target pneumococcal serotype was confirmed, the library containing DNA purified from other streptococci was then utilized as above mentioned to assure reactions did not cross-react with DNA from these nasopharyngeal species.

### Evaluation of long term storage of probes and pre-mixed qPCR reaction cocktails at -20°C

To evaluate proper fluorescence activity of our FAM-labeled qPCR probes, once probes were received in the lab they were solubilized with DNA grade water to a final concentration of 5 μM and utilized immediately, or cryopreserved at -20°C in 100 μl aliquots. Efficiency of qPCR reactions of cryopreserved probes was evaluated at different time points using reaction-specific standards.

Some qPCR reaction mixtures, including those for serotypes 9AV, 9LN, and *lyt*A, were also prepared cryopreserved at -20°C and efficiency of those reactions was evaluated at different times for four months. Quantitative reactions were performed essentially as described earlier.

### PCR reactions to subtype serogroup 6 strains

These reactions were performed as previously described [37, 38] except that we utilized DNA purified from NP samples. Briefly, DNA (2 μl) was used as template in 25 μl PCR reactions containing 50 pmol/μl of each of the pair of primers listed below, 2.5 mmol/l of dNTP, 2.5 μl of 10xPCR buffer (Qiagen), 0.1 μl of Qiagen HotStar Taq polymerase, and molecular biology grade water (Thermo). Reactions specifically target single nucleotide polymorphism within the *wci*P gene and therefore the following primers will amplify a PCR product from: serotype 6A and 6C, primers wciP584gS (5'-ATTTATATATAGAAAAACTGGCTCATGATAG-3') and, wciPr (5'-GCGGAGATAATTTAAAATGATGACTAGTTG-3'), or a PCR product from serotype 6B and 6D with primers wciP584aS (5'-AAGATTATTTATATATAGAAAAACTGTCTCATGATAA-3') and wciPr. Cycling parameters were: one cycle at 95°C for 15 min, 35 cycles of 94°C for 30 s, 62°C for 1 min, and 72°C for 1 min; and a final extension of 72°C for 10 min. Products were run on 3% agarose gels, stained with SYBR Safe DNA gel stain (life technologies, Grand Island, NY) and visualized under a UV transilluminator (BioRad, Hercules CA).

## Results

### Validation, optimization and limit of detection of qPCR assays for detecting pneumococcal serotypes

A total of 27 new serotype/sergroup-specific qPCR assays were developed in this study including 20 serotype-specific reactions and 7 reactions detecting serotypes within a specific serogroup (i.e, 6C and 6D) or two different serogroups. Bioinformatic studies and our high-throughput validation system confirmed the specificity of the new assays for the target pneumococcal serotype(s). They did not cross-react with DNA from nasopharyngeal streptococci and only detected the pneumococcal serotypes listed. The concentration of primer and probe of new qPCR assays (N = 27), and those of available sequences (N = 26), were further optimized for their use in quantitative single-plex reactions and results reported in Table 1 and S1 Table. Together, this panel of newly developed and optimized qPCR assays detect and quantify bacterial loads for most important pneumoccocal serotypes/serogroups. To investigate the limit of detection and efficiency of our quantitative assays, serially diluted DNA was evaluated at least three times using all qPCR reactions. Most reactions were able to detect as little as 5 fg of *S*. *pneumoniae* DNA, the genome equivalent of ∼2 bacteria per reaction, with an efficiency >90% (Table 1 and S1 Table). Quantitative reactions were not able to detect 1 genome equivalent (∼2.5 fg) even after 60 qPCR cycles (not shown).

### Cryogenic preservation of *S*. *pneumoniae* serotype-specific FAM-labeled qPCR probes

A number of fluorescence-labeled probes would be required in order to detect and quantify by qPCR most *S*. *pneumoniae* serotypes. Therefore, we evaluated during a 2-year period the limit of detection and efficiency of 14 selected qPCR reactions whose probes were stored at -20°C protected from light sources. At day 0, probes were diluted with molecular grade DNase-, RNase-free water to a final 5 μM concentration and immediately utilized in qPCR reactions. At the same time 100 μl aliquots were made and stored at -20°C until future testing.

An aliquot of 100 μl of a FAM-labeled probe (5 μM) is enough to run qPCR reactions in two 96-well plates, therefore aliquots were thawed/freeze only once or twice. S2 Table shows that efficiency of reactions containing probe 19BF or probe for serogroup 15 remained >93% after 2 years at -20°C. Most quantitative reactions detected the genome equivalent of 2 bacteria throughout the trial period (S2 Table). Similar results were obtained with other 12 probes including those probes for qPCR assays targeting vaccine serotypes 1, 4, 6ABCD, 9AV, 14 or 23F. Therefore, cryogenic storage of these FAM-labeled probes for nearly two years did not affect the limit of detection of qPCR assays.

### Cryopreservation of premixed qPCR assays stored at -20°C

Preparing reaction mixtures for several qPCR assays might be time-consuming if different targets (i.e., for instance all these serotypes) are pursued. Quantitative molecular assays are also highly sensitive whereby it is relatively easy to contaminate the reaction mixture with *S*. *pneumoniae* DNA if a dedicated room to assemble the reaction mixtures is not available. Therefore, we evaluated the efficiency and limit of detection of selected qPCR assays that had been premixed and cryopreserved at -20°C for up to 2 months. We did not evaluate reaction mixtures stored at -80°C since the qPCR master mix should be kept at -20°C.

The qPCR assay was mixed as described in Materials and Methods and aliquots were made. One of these aliquots was immediately utilized in qPCR reactions while all others were stored at -20°C. Aliquots were then thawed either 1 day, 7 days, 1 month or 2 months later and qPCR reactions were performed. S3 Table shows that all cryopreserved aliquots had a similar high efficiency (≥91.6%) as that of the freshly prepared reaction mixture. Reaction mixtures kept at -20°C for up to a month for *lyt*A, or two months for those mixtures targeting serotype 9AV or 9LN, detected 2.1 genome equivalent per reaction (S3 Table). The *lyt*A reaction mixture was able to detect 21.4 genome equivalent per reaction when cryopreserved for 2 months. Our results indicate that ready-to-use reaction mixtures can be prepared and stored for up to 1 month at -20°C with no loss of efficiency or limit of detection.

### Detection and quantification of bacterial load of pneumococcal serotypes in nasopharyngeal samples

To date, real-time PCR studies for detecting pneumococcal serotypes have focused on no more than 23 different serotypes/serogroups in NP samples. Having validated and optimized 53 single-plex qPCR reactions, we investigated the presence, and quantified the bacterial load, of these serotypes/serogroups in NP samples. For these proof-of-concept studies, we selected NP samples that had been previously screened for the presence of *S*. *pneumoniae* by the *lyt*A qPCR assay. As we were primarily interested in detection of multiple serotypes/serogroups, these NP samples were selected to contain ≥10^6^ CFU/ml of *S*. *pneumoniae*, as we thought high *S*. *pneumoniae* loads might be indicative of colonization by multiple serotypes.

**Fig 1:**
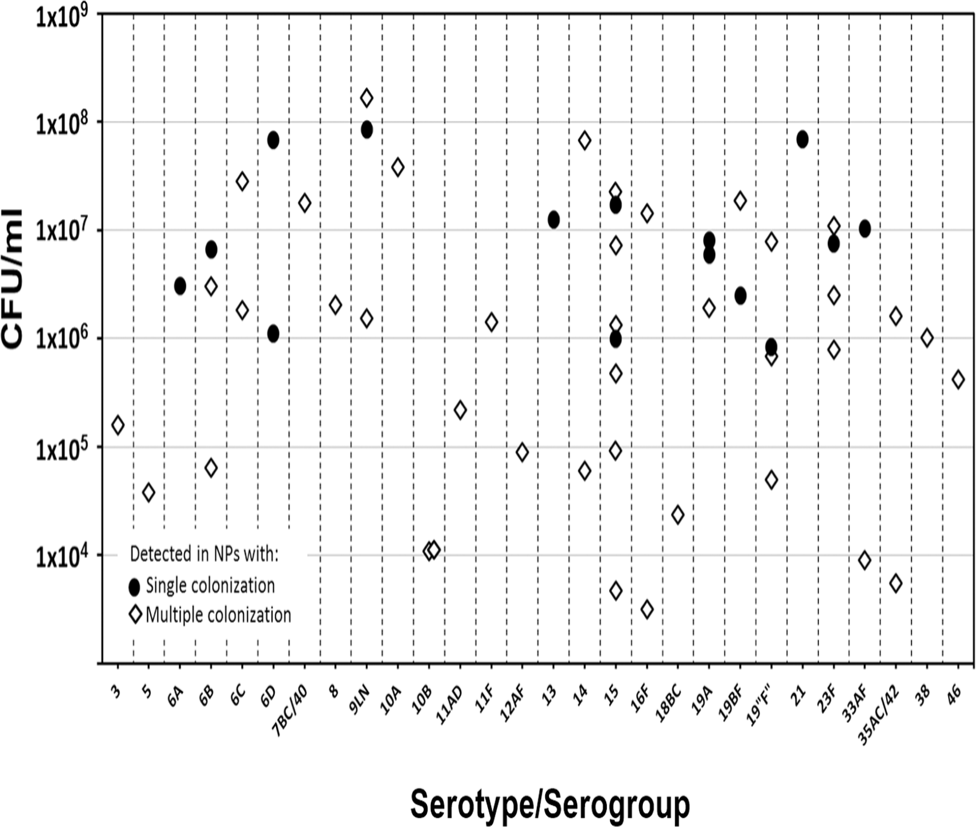
Distribution and densities of *S*. *pneumoniae* serotypes in single or multiple colonized nasopharyngeal (NP) samples. Density (CFU/ml as determined by *lytA* qPCR) of the specified serotype is presented on the x axis. Symbols for each serotype indicates the number of times they were detected as a unique serotype (•) or in samples with multiple colonization (◊).

In contrast to what we hypothesized, our studies demonstrated colonization by a single *S*. *pneumoniae* strain in 50% (N = 15) of these NP samples (not shown). The bacterial load of the identified serotypes (≥10^6^ CFU/ml) correlated with the load of *S*. *pneumoniae* obtained by the *lyt*A assay, further supporting these samples contain only one serotype (Fig. 1, black dots). Strains belonging to serotypes 6D, and 19A and serogroup 15, were detected in two different samples, whereas 6A, 6B, 19BF, 19“F”, 23F, 9LN, 13, 21, and 33AF were detected in only one (Fig. 1). Serotype 13 and serotype 21 in this study were only detected in NPs with single serotype colonization.

Multiple colonization (≥2 serotypes) was observed in 50% of the NP samples tested. Of these, 33% carried 2 different serotypes, 7% contained 3 serotypes, 3% carried 4 serotypes, and 5 serotypes were detected in 7% of samples (not shown). NP samples with multiple colonization always contained a strain that contributed more than 50% of the bacterial load (Table 2). For example, NP specimen 29 contained in proportion 99.94% (1.44x10^7^ CFU/ml; Cq 26.84) of serotype 16F vs only 0.06% (8.93x10^3^ CFU/ml; Cq 38.0) of serotype 33A/F (Table 2). A total of 11 different serotypes/serogroups were carried as proportionally dominant (>50% of the total load). Eleven serotypes/serogroups were only carried at a low proportion (<50%) including vaccine serotype 6B and emergent 6C. Some strains, such as those of serogroup 15, contributed in proportion with as low as 0.02% (4.7x10^3^ CFU/ml; Cq 38.7) of total pneumococcal load and as high as 99.3% (2.2x10^7^ CFU/ml; Cq 25.8) while these strains were also detected in samples containing, 2, 3, 4 or 5 different serotypes (Table 2 and S4 Table). The highest bacterial load of a given serotype/serogroup in NP samples containing multiple serotypes/serogroups was 2.1x10^8^ CFU/ml of serotype 9 in a NP sample containing 2 strains whereas the lowest load detected was 3.15x10^3^ CFU/ml of serotype 16F (Cq 39.4) in a NP sample containing 5 different serotypes/serogroups (S4 Table).

**Table 2:** Proportion of pneumococcal serotypes quantified by qPCR in nasopharyngeal samples containing multiple serotypes.

Sample ID	Total Sp load*CFU/ml	number of serotypes detected	Proportion (%) of serotypes																							
			3	5	6B	6C	7BC/40	8	9LN	10A	10B	11AD	11F	12AF	14	15	16F	18BC	19A	19BF	19“F”	23F	33AF	35AC/42	38	46
9	1.67E+08	5				21.19				28.69	0.01				50.10			0.02								
19	2.37E+07	5												0.46		0.02	0.02			97.33						2.17
6	1.12E+07	4				20.11					0.12					79.22					0.54					
11	1.45E+07	3											10.33			9.70						79.97				
5	3.47E+06	3							43.92						1.72				54.37							
7	1.30E+08	2							99.72							0.28										
28	4.34E+07	2	0.70													99.30										
10	1.52E+07	2		0.48																	99.52					
14	1.23E+07	2			14.35		85.65																			
29	3.90E+06	2															99.94						0.06			
18	2.51E+06	2						55.56																44.44		
16	2.39E+06	2																						0.53	99.47	
3	1.86E+06	2			2.51																	97.49				
23	1.47E+06	2										21.60										78.40				
8	1.05E+06	2														11.80					88.20					

*Calculated using the *lyt*A assay.

### Comparison of serotyping results by culture and by qPCR, including correlation between the dominant serotype identified by each method

Serotyping results obtained by qPCR were compared with results from previous analysis by culture and traditional serotyping of up to 120 colonies using a combination of latex agglutination and the Quellung reaction [28], with the numerically dominant serotypes obtained by culture compared with those proportionally dominant by qPCR (Table 3). In samples containing a single serotype/serogroup (n = 14), there was a perfect correlation between the dominant serotype obtained by culture to that obtained by qPCR, except for one culture-negative sample (NP4), in which serotype 6D was identified by qPCR. In samples with multiple colonization (n = 15), the dominant serotype obtained by culture correlated with the serotype/serogroup present in highest proportion as quantified by qPCR only in ∼73% (n = 11) samples (Table 3). In the remaining ∼33% (n = 4), the dominant serotype isolated by culture did not correlate with the proportionally dominant type by qPCR but could be detected in most samples at a lower proportion. In the samples with multiple colonization, a total of 40 serotypes were identified by qPCR, of which 23 (57.5%) were also identified by culture. There were three serotypes which were identified by culture and not qPCR: serotype 3 in NP23, serotype 11A in NP11 (although the closely related 11F was detected by qPCR, suggesting a possible genetic variant), and serotype 17F in NP6 (not tested by qPCR).

**Table 3:** Agreement between serotypes obtained by culture and those obtained by qPCR.

	Serotypes detected by culture		Serotypes detected by qPCR	
NP	Dominant serotype	Other serotypes*	Dominant serotype	Other serotypes
9	6C	10A, NT	14	10A, 6C, 18BC, 10B
19	19B		19BF	46, 12AF, 15, 16F
6	15A	6C, 17F	15	6C, 19“F”,10B
11	11A	15B/C, 23F	23F	11F, 15
5	19A	9L, 14	19A	9LN, 14
7	9L		9LN	15
28	15A		15	3
10	19F	NT	19“F”	5
14	7B		7B/40	6B
29	16F		16F	33AF
18	35A	8	8	35AC/42
16	25A/38		38	35AC/42
3	23F		23F	6B
23	3	23F, 11A	23F	11AD
8	19F	15C	19“F”	15
4	none		6D	
1	21		21	
26	9L		9LN	
12	15B		15	
21	19A		19A	
24	19A		19A	
25	6B		6B	
22	23F		23F	
20	6A		6A	
30	13		13	
2	19F		19BF	
27	33A		33AF	
15	15B		15	
13	6D		6D	
17	19F		19“F”	

* NT = non-typeable pneumococci

### A molecular approach, S6-q(PCR)^2^, to identify serogroup 6 strains directly in NP samples

**Fig 2:**
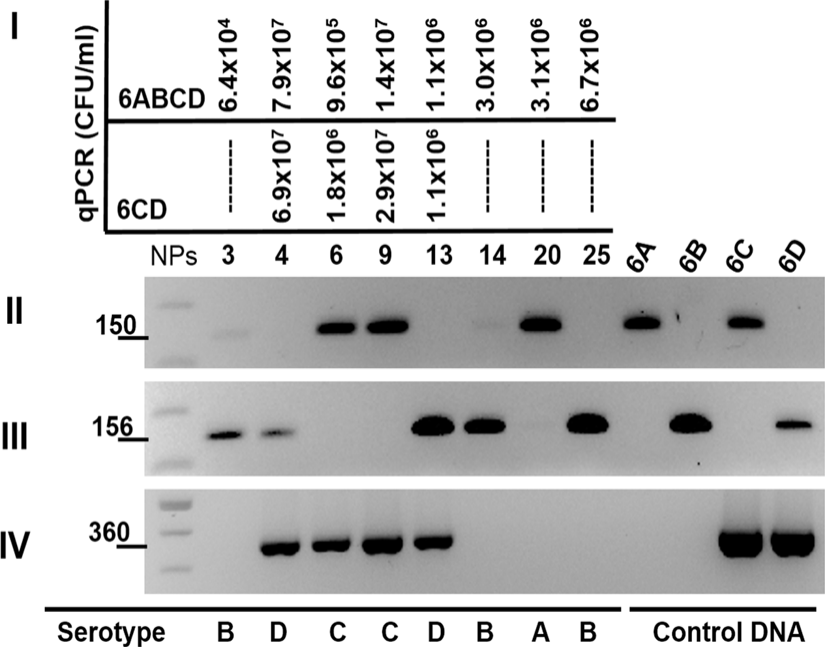
Quantification and subtyping, by exclusion, of serotypes 6A, 6B, 6C, and 6D by S6-q(PCR)^2^. DNA from NP samples was utilized as template in qPCR and PCR reactions. (I) The first set of qPCR reactions quantified serogroup 6, including serotype 6A, 6B, 6C and 6D (top panel), whereas the second specifically quantified serotypes 6C and 6D (bottom panel). Serotype 6A and 6B or serotype 6C or 6D are differentiated by one single nucleotide polymorphisms (SNPs) in the *wci*P gene. To type individual serotypes, PCR reactions were performed targeting subtype SNPs within the *wci*P gene to identify (II) serotypes 6A and 6C or (III) serotypes 6B and 6D strain. A PCR targeting (IV) serotypes 6C and 6D specifically by amplifying the *wci*Nβ gene was performed as an additional control.

Some qPCR assays are unable to differentiate among closely-related specific serotypes, such as those within serogroup 6. To begin exploring alternative approaches, we designed a molecular procedure that allowed us to individually type serogroup 6 strains directly from NP swabs. As this procedure includes a series of qPCR and PCR reactions targeting serogroup 6, it was named S6-q(PCR)^2^. This series of reactions will type individual serotypes belonging to serogroup 6 by exclusion while obtaining the bacterial density of each serotype. In a first step, a qPCR reaction detects serogroup 6 (i.e., 6A, 6B, 6C, and 6D), and a second reaction specifically targets serotypes 6C and 6D. Our qPCR studies thus revealed that 8 out of 30 NP samples contained serogroup 6 strains, with 4 of them giving a positive reaction for 6C and 6D [Fig. 2 panel (I)]. These results indicate that NPs 4, 6, 9, and 13 carry either 6C or 6D and, by exclusion, the other 4 NP samples contain 6A or 6B (e.g., NPs 3, 14, 20, and 25).

**Fig 3:**
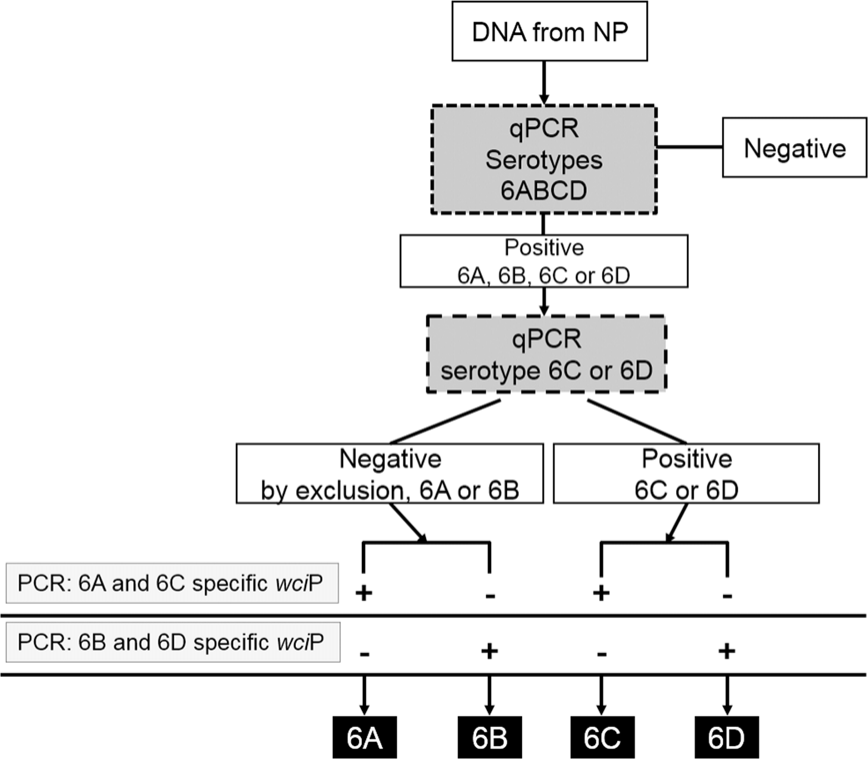
Algorithm for the identification and quantification of serotype 6 types directly from NP samples by S6-q(PCR)^2^. DNA purified from NP samples is utilized as template in qPCR and PCR reactions. Arrows indicate reactions to be performed, qPCR in dark gray boxes or PCR in light gray boxes, and continuous lines indicate reaction results. The final serotype obtained is indicated in the black boxes.

Having separated NPs into those containing 6A or 6B or those with 6C or 6D, to identify individual serotypes we utilized two different conventional PCR reactions. These reactions target nucleotide polymorphisms within the *wci*P gene, thereby detecting the presence of 6A and 6C or 6B and 6D [37, 38]. As we have reported that DNA from NP samples containing ≥10^4^ CFU/ml of *S*. *pneumoniae* load generates an amplicon by conventional PCR [31], extracted DNA from NP samples positive for serogroup 6 by qPCR was used as template. As hypothesized, serotype 6-specific PCR products were amplified from all NPs (Fig. 2 panels II, III, and IV). The first PCR reaction identified three NPs with serotype 6A or 6C strains, and therefore NP sample 20, which had been typed by qPCR as carrying 6A or 6B, contains serotype 6A. Utilizing the same algorithm, NPs 6 and 9 that had been typed by qPCR to have 6C or 6D, carried serotype 6C strains [Fig. 2, panel (II)]. As expected, the second PCR reaction that targets 6B and 6D was negative for these three NPs. In this second reaction, the other five NPs were positive indicating that NPs 3, 14, and 25 contained serotype 6B while NPs 4 and 13 carried a serotype 6D strain [Fig. 2, panel (II) and (III)]. The last reaction [Fig. 2, panel (IV)], identifies serotypes 6C or 6D and served as an extra control for our studies. Fig. 3 shows a summary of our molecular algorithms for the identification and quantification of serotype 6 strains utilizing S6-q(PCR)^2^. This molecular approach proved to be useful to identify, and quantify serogroup 6 strains directly in DNA purified from NP specimens.

## Discussion

In this study we have optimized and validated a total of 53 qPCR assays, including 27 newly developed assays, for the detection and quantification of the bacterial load for pneumococcal serotypes/serogroups. This represents an important contribution to the currently available 12 [39], 16 [40] or 21 [27] multiplex qPCR reactions for *S*. *pneumoniae* serotypes/serogroups. These new qPCR assays can be utilized to expand currently available multiplex assays. Multiplex reactions for most pneumococcal serotypes can be first utilized to identify serotypes/serogroups in field samples. Once the serotype(s) is identified, then single-plex qPCR reactions optimized in this study will allow us to obtain the specific bacterial load.

Using these new qPCR assays (Table 1), our proof-of-concept studies identified multiple serotypes/serogroups in a number of samples examined, and the bacterial load was determined. Similar studies have identified more than five pneumococcal serotypes using real-time PCR or microarrays in specimens collected from Dutch or Thai children, respectively [19, 41]. Our studies, however, were limited to a small number of samples. Future studies with a larger number of specimens are warranted. While identifying serotypes by using multiplexed reactions may be an appropriate approach for field studies, having single-plex reactions able to quantify the bacterial density may also be required as efforts are being made to utilize nasopharyngeal density to diagnose pneumococcal pneumonia [42]. Density may also be an important measure of vaccine impact. Carriage density may predict likelihood of transmission and impact herd immunity. For example, in HIV-infected adults hospitalized with radiographically confirmed community acquired pneumonia (CAP), a *lyt*A qPCR density ≥8000 CFU/ml had a sensitivity of 82.2% and a specificity of 92.0% for distinguishing pneumococcal CAP from asymptomatic colonization [42].

Most quantitative assays for pneumococcal serotypes developed here (N = 21) detected ∼2.1 genome equivalent per reaction whereas for the remaining 6 assays the limit of detection was ∼21.4 genome equivalent per reaction. This represents an improved limit of detection or similar limit of detection, respectively, to that reported earlier [27, 39, 40].

Quantitative assays reported in this study were validated in reactions containing a final volume of 10 μl, in contrast to all other published reactions that utilize 25 μl of final reaction volume [22, 27, 39, 40]. Reducing the reaction volume also allowed us to reduce the volume of DNA template without compromising the limit of detection. For comparison purposes, other studies have utilized 2 [39], 2.5 [40], 5 [27] or 6 [22] μl of DNA template whereas our validated protocol requires only 1 μl per reaction. Reducing the amount of DNA template might allow the use of these reactions in retrospective studies where the volume left from stored biological material is an important issue.

This report is to the best of our knowledge the first to demonstrate that 2-years cryopreserved, FAM-labeled, qPCR probes yielded a similar reaction efficiency as that of freshly prepared probes. This information is important for planning and budgetary purposes when conducting longitudinal studies as sets of primers and probes do not need to be re-stocked within 2 years. Moreover, the current availability of real-time systems almost everywhere in the world but the lack of appropriate laboratory facilities to prepare qPCR reaction mixtures avoiding contamination (i.e., a dedicated room with laminar flow hood for mixing components of the qPCR assays, etc.) motivated us to test the stability at -20°C of qPCR reaction mixtures. There was no loss of limit of detection in reaction mixtures cryopreserved for at least 1 month, thereby studies involving different countries or different locations within the same country may benefit by preparing the mixtures in central facilities, and then distributing ready-to-use real-time reagents. Additionally, molecular testing with the exact same reagent may generate more consistent results throughout study sites.

As mentioned earlier, the aim of this study was not to conduct an epidemiological investigation, but to make the primers and probe sequences and reaction protocols available while testing NP samples as a proof-of-concept of what the new assays could add to the field. The density of the total pneumococcal load (Table 2) did not appear to indicate the presence of single or multiple colonization, since four samples containing only one serotype (NPs 1, 4, 12 and 26) had loads higher than one of the two samples containing 5 different serotypes (NP 19, 2.4x10^7^ CFU/ml). As expected, in samples where only one serotype was found, the serotype result by qPCR correlated to that obtained by culture. In contrast, in samples containing multiple serotypes, the dominant serotype by culture did not always match that present in the highest proportion by qPCR, and many serotypes detected by qPCR were not found using culture-based methods. These discrepancies may be due to a variety of reasons, including loss of viability following freeze-thaw, increased limit of detection of the qPCR for detecting serotypes present in low numbers, or subtle differences between the aliquots tested.

Atypical serotype 19F strains (referred here as 19“F”) were detected and quantified using a new qPCR reaction. This 19“F” variant was originally reported by Pimenta, et al. (2009) [35]. Authors determined that 19“F” encoded a *wzy* gene (target for molecular typing) with ∼88% identity to known 19A cps loci. Similar strains have been recently isolated in Brazil [36]. Our studies obtained in culture pneumococci that were Quellung typed as 19F (Table 3) but that our qPCR assays targeting serotypes 19F, or 19A, did not detect. A new qPCR reaction designed based on the sequence of the *wzy* gene encoded by those isolates allowed us the identification of 19“F” strains in four nasopharyngeal specimens. This reaction should be helpful for surveillance studies using qPCR platforms for molecular serotyping.

Although qPCR assays were tested against a panel of non-pneumococcal streptococci with no cross-reactivity observed, further confirmation of the specificity of these assays is warranted. For example, Carvalho *et al* (2013) detected positivity for reactions amplifying some pneumococcal serotypes (i.e., 12F/12A/44/46 and 33F/33A/37) in *lyt*A negative nasopharyngeal/oropharyngeal samples and from a *S*. *oralis* strain isolated from those NPs [43]. Our proof-of-concept studies with NP swabs, presented in Table 3, were conducted on *lyt*A positive specimens. Whereas we obtained positivity in a *lyt*A-positive NP specimen for our reaction amplifying serotypes 12AF, from which the strain was not recovered in culture (NP#19); moreover, our reaction targeting 33AF sequences yielded a positive result and a pneumococcal strain serotype 33A was isolated from the NP specimen #27 (Table 3).

Another important contribution within this work is the procedure that we have called S6-q(PCR)^2^ for identifying and quantifying serogroup 6 strains. There is currently no available real-time assays to identify and quantify individual serogroup 6 serotypes directly from NP swabs. Available PCR assays require the isolation of the serotype by culture and use purified DNA as template [37, 38]. Detecting specific serotypes within serogroup 6 strains is particularly important as serotypes 6A and 6B are vaccine types with a high prevalence worldwide in PD [9, 11]. Furthermore, 6C strains are increasing in prevalence post PCV introduction [44–46], while 6D strains have recently emerged as a new serotype [37, 47, 48]. Strains carrying polymorphisms within the *wzy* gene belonging to a variant called “6E” have been isolated in Korea; whether these strains exist in other countries is unknown [49]. We combined two technologies, qPCR and conventional PCR, to successfully identify and quantify individual serogroup 6 types in 8 different NP samples. S6-q(PCR)^2^ required two qPCR reactions. The first reaction identified samples containing serogroup 6 (6A, 6B, 6C or 6D) and obtained the density. A second qPCR reaction separated samples with serotypes 6C, 6D or by exclusion, if the reaction was negative, serotype 6A or 6B. Finally, conventional PCR dissected the presence of specific types (Fig. 3). S6-q(PCR)^2^ identified vaccine type 6B and 6D in NPs where they had not been identified by culture (Table 3). Interestingly, the bacterial load of 6B or 6C strains in NP colonized by multiple serotypes was proportionally low in comparison with the dominant serotype. If this is happening in PCV-vaccinated populations, then examination of serotypes by culture-based methods could under-report these important serotypes. A limitation of S6-q(PCR)^2^ is that the PCR component of the scheme requires a bacterial density of ∼1x10^4^ CFU/ml to generate a PCR amplicon.

The introduction of pneumococcal conjugate vaccines more than 10 years ago have considerably reduced the burden of pneumococcal diseases in countries were children have been vaccinated. In contrast, NP carriage of the pneumococcus have been maintained at similar rates since serotypes not included in the vaccine have now gained access to, or increased in density in the nasopharynx. The future of epidemiological and clinical studies to evaluate vaccination efficacy may require both highly sensitive and quantitative assays.

## Supporting Information

S1 Table Concentration of primers and probes of quantitative assays optimized in this study. (DOCX)

S2 Table Efficiency and limit of detection of cryopreserved probes. (DOCX)

S3 Table Efficiency and limit of detection of cryopreserved, ready-to-use qPCR reaction mixtures. (DOCX)

S4 Table Bacterial load and Cq values obtained in qPCR studies. (DOCX)
