# Supplementary material for: Single-Plex Quantitative Assays for the Detection and Quantification of Most Pneumococcal Serotypes
Source: PLoS One. 2015 Mar 23;10(3):e0121064. doi: 10.1371/journal.pone.0121064 (PMC4370668; doi:10.1371/journal.pone.0121064)
Supplement: S1 Table — (DOCX) [file pone.0121064.s001.docx]

**S1_Table. Concentration of primers and probes of quantitative assays optimized in this study.**

| Serotype/serogroup* | Primer (nM) | Probe (nM)** | Limit of detection (fg) | Reference |
| --- | --- | --- | --- | --- |
| 1 | 500 | 500 (JOE) | 50 | ([1](#_ENREF_1)) |
| 3 | 400 | 200 | 5 | ([1](#_ENREF_1)) |
| 4 | 300 | 200 | 50 | ([1](#_ENREF_1)) |
| 5 | 500 | 500 (JOE) | 50 | ([1](#_ENREF_1)) |
| 6ABCD | 500 | 200 | 5 | ([1](#_ENREF_1)) |
| 7AF | 200 | 200 | 5 | ([1](#_ENREF_1)) |
| 8 | 500 | 500 (JOE) | 50 | ([1](#_ENREF_1)) |
| 9VA | 500 | 200 | 5 | ([1](#_ENREF_1)) |
| 11AD | 400 | 200 | 5 | ([2](#_ENREF_2)) |
| 12ABF | 200 | 200 | 5 | ([1](#_ENREF_1)) |
| 14 | 400 | 200 | 5 | ([1](#_ENREF_1)) |
| 15ABCF | 400 | 200 | 5 | ([1](#_ENREF_1)) |
| 16F | 400 | 200 | 5 | ([2](#_ENREF_2)) |
| 18BC | 400 | 200 | 5 | ([1](#_ENREF_1)) |
| 19A | 400 | 200 | 5 | ([1](#_ENREF_1)) |
| 19BF | 400 | 200 | 5 | ([1](#_ENREF_1)) |
| 20 | 500 | 400 | 5 | ([1](#_ENREF_1)) |
| 21 | 500 | 300 | 5 | ([2](#_ENREF_2)) |
| 22AF | 400 | 200 | 50 | ([1](#_ENREF_1)) |
| 23A | 400 | 200 | 5 | ([2](#_ENREF_2)) |
| 23B | 400 | 200 | 50 | ([2](#_ENREF_2)) |
| 23F | 300 | 200 | 5 | ([1](#_ENREF_1)) |
| 29 | 400 | 200 | 5 | ([2](#_ENREF_2)) |
| 33AF | 400 | 200 | 50 | ([1](#_ENREF_1)) |
| 35B | 400 | 200 | 5 | ([1](#_ENREF_1)) |
| 38 | 400 | 400 | 5 | ([1](#_ENREF_1)) |

*Sequences are listed in the associated reference.

**Probes were labeled at 5’ with FAM, except where indicated (JOE^TM^), and at 3’ with BHQ1.

**S1_Table, references**

1. **Azzari C, Moriondo M, Indolfi G, Cortimiglia M, Canessa C, Becciolini L, et al.**  2010. Realtime PCR Is More Sensitive than Multiplex PCR for Diagnosis and Serotyping in Children with Culture Negative Pneumococcal Invasive Disease. PLoS One. **5(2): e9282. doi:10.1371/journal.pone.0009282.**

2. **Azzari C, Moriondo M, Cortimiglia M, Valleriani C, Canessa C, Indolfi G, et al.**  2012. Potential serotype coverage of three pneumococcal conjugate vaccines against invasive pneumococcal infection in Italian children. Vaccine **30:**2701-2705.
