# Supplementary material for: Single-Plex Quantitative Assays for the Detection and Quantification of Most Pneumococcal Serotypes
Source: PLoS One. 2015 Mar 23;10(3):e0121064. doi: 10.1371/journal.pone.0121064 (PMC4370668; doi:10.1371/journal.pone.0121064)
Supplement: S2 Table — (DOCX) [file pone.0121064.s002.docx]

**S2_Table. Efficiency and limit of detection of cryopreserved probes**

| Serotype | Date | Efficiency (%) | Limit of detection (fg) | Genome equivalent (Copy number) |
| --- | --- | --- | --- | --- |
| 1 | January, 2012 | 97.6 | *50 | 21.4 |
|  | February, 2014 | 93.0 | **50 | 21.4 |
| 4 | January, 2012 | 98.9 | *50 | 21.4 |
|  | April, 2014 | 97.4 | **50 | 21.4 |
| 5 | January, 2012 | 98.2 | *50 | 21.4 |
|  | September, 2012 | 95.6 | *50 | 21.4 |
|  | April, 2013 | 93.5 | 5 | 2.1 |
|  | February, 2014 | 96.0 | *50 | 21.4 |
| 6ABCD | December, 2011 | 90.1 | *50 | 21.4 |
|  | September, 2012 | 92.1 | *50 | 21.4 |
|  | February, 2014 | 96.1 | 5 | 2.1 |
| 8 | January, 2012 | 98.7 | *50 | 21.4 |
|  | September, 2012 | 100.2 | *50 | 21.4 |
|  | February, 2014 | 95.6 | *50 | 21.4 |
| 9VA | December, 2011 | 92.4 | *50 | 21.4 |
|  | October, 2012 | 99.4 | *50 | 21.4 |
|  | May, 2013 | 90.2 | 5 | 2.1 |
|  | February, 2014 | 98.1 | 5 | 2.1 |
| 12ABF | January, 2012 | 98.0 | *50 | 21.4 |
|  | February, 2014 | 102.5 | 5 | 2.1 |
| 14 | December, 2011 | 93.6 | *50 | 21.4 |
|  | September, 2012 | 92.1 | *50 | 21.4 |
|  | April, 2013 | 90.9 | 5 | 2.1 |
|  | February, 2014 | 97.3 | 5 | 2.1 |
| 15 | December, 2011 | 94.2 | *50 | 21.4 |
|  | October, 2012 | 92.4 | *50 | 21.4 |
|  | May, 2013 | 92.9 | 5 | 2.1 |
|  | March, 2014 | 92.9 | 5 | 2.1 |
| 19BF | January, 2012 | 97.0 | *50 | 21.4 |
|  | May, 2013 | 99.2 | 5 | 2.1 |
|  | May, 2013 | 92.8 | 5 | 2.1 |
|  | August, 2013 | 97.3 | 5 | 2.1 |
|  | February, 2014 | 98.8 | 5 | 2.1 |
| 20 | January, 2012 | 91.7 | *50 | 21.4 |
|  | March, 2014 | 93.8 | 5 | 2.1 |
| 23F | February, 2012 | 96.6 | *50 | 21.4 |
|  | May, 2013 | 98.7 | 5 | 2.1 |
|  | February, 2014 | 98.1 | 5 | 2.1 |
| 29 | January, 2012 | 107.8 | *50 | 21.4 |
|  | May, 2013 | 91.2 | 5 | 2.1 |
|  | February, 2014 | 98.2 | 5 | 2.1 |
| 38 | January, 2012 | 100.0 | *50 | 21.4 |
|  | June, 2013 | 99.1 | **50 | 21.4 |
|  | March, 2014 | 101.9 | 5 | 2.1 |

*5 fg were not tested. **5 fg were tested
