# Supplementary material for: Single-Plex Quantitative Assays for the Detection and Quantification of Most Pneumococcal Serotypes
Source: PLoS One. 2015 Mar 23;10(3):e0121064. doi: 10.1371/journal.pone.0121064 (PMC4370668; doi:10.1371/journal.pone.0121064)
Supplement: S3 Table — (DOCX) [file pone.0121064.s003.docx]

**S3_Table. Efficiency and limit of detection of cryopreserved, ready-to-use qPCR reaction mixtures**

| qPCR reaction mixture | Utilized at: | Efficiency (%) | Limit of detection (fg) | Genome equivalent (copy number) |
| --- | --- | --- | --- | --- |
| *lyt*A | day 0 | 93.9 | 5 | 2.14 |
|  | 1 month | 91.6 | 5 | 2.14 |
|  | 2 month | 96.4 | 50 | 21.4 |
| 9VA | day 0 | 108.9 | 5 | 2.14 |
|  | 1 month | 104 | 5 | 2.14 |
|  | 2 month | 96.7 | 5 | 2.14 |
| 9LN | day 0 | 102.4 | 5 | 2.14 |
|  | 1 month | 104.8 | 5 | 2.14 |
|  | 2 month | 97.4 | 5 | 2.14 |
