# Supplementary material for: Single-Plex Quantitative Assays for the Detection and Quantification of Most Pneumococcal Serotypes
Source: PLoS One. 2015 Mar 23;10(3):e0121064. doi: 10.1371/journal.pone.0121064 (PMC4370668; doi:10.1371/journal.pone.0121064)
Supplement: S4 Table — (DOCX) [file pone.0121064.s004.docx]

|  | **Serotypes detected by qPCR and bacterial load (CFU/ml; Cq)** | |
| --- | --- | --- |
| **NP** | **Dominant serotype** | **Other serotypes** |
| 9 | 14 (6.7x10^7^; 25.8) | 10A (3.9x10^7^; 25.1), 6C (2.9x10^7^; 24.3), 18BC (2.4x10^4^; 36.3), 10B (1.2x10^4^; 38.0) |
| 19 | 19BF (1.9x10^7^; 27.0) | 46 (4.2x10^5^; 32.4), 12AF (8.9x10^4^; 33.8), 15 (4.7x10^3^; 38.8), 16F (3.2x10^3^; 39.4) |
| 6 | 15 (7.2x10^6^; 27.8) | 6C (1.8x10^6^; 28.2), 19“F” (5.0x10^4^; 34.8), 10B (1.1x10^4^; 38.1) |
| 11 | 23F (1.1x10^7^; 25.8) | 11F (1.4x10^6^; 28.0), 15 (1.3x10^6^; 30.2) |
| 5 | 19A (1.9x10^6^; 29.0) | 9LN (1.5x10^6^; 29.8), 14 (6.0x10^4^; 36.0) |
| 7 | 9LN (1.7x10^8^; 23.1) | 15 (4.7x10^5^; 31.8) |
| 28 | 15 (2.3x10^7^; 25.9) | 3 (1.6x10^5^; 33.2) |
| 10 | 19“F” (7.9x10^6^; 27.7) | 5 (3.8x10^4^; 35.7) |
| 14 | 7B/40 (1.8x10^7^; 25.0) | 6B (3.0x10^6^; 27.4) |
| 29 | 16F (1.4x10^7^; 26.8) | 33AF (8.9x10^3^; 38.0) |
| 18 | 8 (2.0x10^6^; 27.3) | 35AC/42 (1.6x10^6^; 30.9) |
| 16 | 38 (1.0x10^6^; 31.1) | 35AC/42 (5.5x10^3^; 39.5) |
| 3 | 23F (2.5x10^6^; 28.0) | 6B (6.4x10^4^; 33.2) |
| 23 | 23F (7.9x10^5^; 29.6) | 11AD (2.2x10^5^; 31.0) |
| 8 | 19“F” (6.9x10^5^; 31.0) | 15 (9.2x10^4^; 34.3) |
| 4 | 6D (6.9x10^7^; 23.1) | ND* |
| 1 | 21 (6.9x10^7^; 24.6) | ND |
| 26 | 9LN (8.5x10^7^; 24.1) | ND |
| 12 | 15 (1.7x10^7^; 26.3) | ND |
| 21 | 19A (8.0x10^6^; 27.1) | ND |
| 24 | 19A (6.0x10^6^; 27.4) | ND |
| 25 | 6B (6.7x10^6^; 26.3) | ND |
| 22 | 23F (7.6x10^6^; 26.3) | ND |
| 20 | 6A (3.1x10^6^; 27.4) | ND |
| 30 | 13 (1.3x10^7^; 28.2) | ND |
| 2 | 19BF (2.5x10^6^; 30.3) | ND |
| 27 | 33AF (1.0x10^7^; 27.4) | ND |
| 15 | 15 (1.0x10^6^; 30.6) | ND |
| 13 | 6D (1.1x10^6^; 28.8) | ND |
| 17 | 19“F” (8.4x10^5^; 30.7) | ND |

**S4_Table. Bacterial load and Cq values obtained in qPCR studies.**

*ND, additional strains were not detected.
